# Rituximab with high-dose methotrexate is effective and cost-effective in newly diagnosed primary central nervous system lymphoma

**DOI:** 10.1038/s41598-022-24922-y

**Published:** 2022-12-13

**Authors:** Xianggui Yuan, Teng Yu, Yurong Huang, Huawei Jiang, Xiaohua Xu, Yun Liang, Wenbin Qian

**Affiliations:** 1 Department of Hematology, the Second Affiliated Hospital, Zhejiang University School of Medicine, #1511 Jianghong Road, Hangzhou, 310058 China; 2 National Clinical Research Center for Hematologic Diseases, the First Affiliated Hospital of Soochow University, Suzhou, China

**Keywords:** Cancer, Medical research, Oncology

## Abstract

Induction chemotherapy based on high-dose methotrexate is considered as the standard approach for newly diagnosed primary central nervous system lymphomas (PCNSLs). However, the best combination chemotherapeutic regimen remains unclear. This study aimed to determine the efficacy and toxicities of rituximab with methotrexate (R-M regimen). Consecutive 37 Chinese patients receiving R-M regimen as induction chemotherapy were retrospectively identified from January 2015 to June 2020 from our center in eastern China. Fourteen patients receiving rituximab plus methotrexate with cytarabine (R-MA regimen) at the same period were identified as the positive control group. The response rates, survival, toxicities, length of hospital stay (LOS), and cost were compared. Compared with the R-MA regimen, the R-M regimen showed comparable response rate and survival outcomes, but had fewer grade 3–4 hematological toxicities, shorter LOS, lower mean total hospitalization cost and lower mean total antibiotic cost. Complete remission at the end of induction chemotherapy and ECOG > 3 were independent prognostic factors for overall survival. In conclusion, R-M regimen is an effective and cost-effective combination treatment for PCNSLs, which warrants further evaluation in randomized trials.

## Introduction

Primary central nervous system lymphoma (PCNSLs) is a rare and aggressive extranodal non-Hodgkin lymphoma located in the brain, leptomeninges, spinal cord, eyes or cerebrospinal fluid (CSF)^1^. PCNSLs are characterized by short-lasting responses and poor prognosis. High-dose methotrexate (HD-MTX) is the standard treatment for newly diagnosed PCNSLs. However, the efficacy of HD-MTX monotherapy is unsatisfactory with a median overall survival (OS) of 16–38 months and progression-free survival (PFS) of 4–10 months^2–4^. As such, other drugs in combination with HD-MTX have been investigated to improve the outcome^5^. Cytarabine (Ara-C) is a cell cycle-phase specific antimetabolite, commonly used in chemotherapy for hematologic malignancies. IELSG20, a phase 2 trial, indicated that the combination of Ara-C with HD-MTX increased the CR rate (46% vs. 18%) and improved the 3-year OS (46 vs. 32%) in PCNSLs^4^. IELSG32, another phase 2 trial, revealed that patients receiving the HD-MTX plus Ara-C (MA regimen) together with thiotepa and rituximab (MATRix) had a higher CR rate (49 vs. 23%) and improved PFS and OS, than those receiving the MA regimen alone^6^. Although combination chemotherapeutic regimens bring increasing efficacy, hematological toxicities are the primary concerns. Therefore, the best combination chemotherapeutic regimen remains unclear.

Rituximab, a highly specific monoclonal antibody against CD20, is widely used to treat diffuse large B cell lymphoma (DLBCL). The combination of rituximab with the cyclophosphamide, doxorubicin, vincristine and prednisone (R-CHOP) regimen has been proved to significantly improve the OS of systemic DLBCL^7^. Although rituximab’s clinical effect in PCNSLs remains controversial due to its large size and poor penetration of the blood–brain barrier (BBB), several preliminary studies suggest that adding rituximab to cytotoxic chemotherapy is safe and effective for PCNSLs^2,8,9^. Studies have indicated that rituximab is likely to reach therapeutic concentrations and can induce responses in contrast-enhanced lesions, in which there is a substantial disruption of the BBB^10,11^. Two randomized controlled trials were designed to formally determine the effects of rituximab on PCNSLs. The IELSG32 study stated that the addition of rituximab to the MA regimen (R-MA regimen) was more likely to induce a response than the MA regimen alone (ORR,74 vs. 53%) and there was a trend for improved PFS (*p* value 0.051) and OS (*p* value 0.095) with overall minimal added toxicities^6^. However, the HOVON 105/ALLG NHL 24 study, a phase 3 randomized study, did not find significantly increased PFS, or OS from the addition of rituximab to the MTX, carmustine, teniposide, and prednisolone (MBVP) chemotherapy regimen compared with the MBVP regimen alone in newly-diagnosed PCNSLs^12^, which was contrary to the results in the IELSG32 study. Interestingly, subgroup analysis in the HOVON105 study showed that the application of rituximab might benefit patients under 60 years of age. Even with the conflicting results of the above two prospective randomized studies, given the relatively low toxicities and single-agent activity of rituximab in relapsed/refractory PCNSLs, rituximab has been widely used in clinical practice and incorporated into clinical trials^13^.

To balance therapy intensification with side effects in our center, rituximab with methotrexate (R-M regimen) is widely applied for PCNSLs. This study aimed to retrospectively evaluate the efficacy and toxicities of the R-M regimen as induction chemotherapy for newly diagnosed PCNSLs.

## Patients and methods

### Patients

From January 2015 to June 2020, consecutive patients with newly diagnosed PCNSLs, receiving the R-M regimen as induction therapy at the Second Affiliated Hospital, Zhejiang University, were identified from medical records. Given the inaccessibility of thiotepa in China, the R-MA regimen but not the MATRix regimen was widely applied since the IELSG32 results was first published in 2016. Thus, patients receiving the R-MA regimen at the same period were identified as the positive control group. Patients fulfilling the following criteria were enrolled in the study: newly diagnosed patients with PCNSL, definite pathological diagnosis of DLBCL, and measurably enhanced lesions for response assessment. Patients with isolated ocular lymphomas or HIV- associated lymphoma were excluded. Informed consent was obtained from all patients enrolled in this study. According to the updated WHO classifications, the diagnosis of PCNSLs was confirmed by two pathologists and consensus was reached for each patient. This study was approved by the Ethics Committee of the Second Affiliated Hospital, Zhejiang University. All research was performed in accordance with relevant guidelines/regulations in accordance with the Declaration of Helsinki. Informed consent was obtained from all participants and/or their legal guardians.

### Treatment protocol

Patients were scheduled to receive 6 cycles of induction chemotherapy on a 21-day cycle. Thereafter, consolidation treatment with whole-brain radiation therapy (WBRT, 30–36 Gy followed by a limited field to gross disease to 45 Gy) was administered, depending on their age, financial situation and willingness. Patients who failed to reach PR after 4 courses of induction chemotherapy or progressed at any time were withdrawn and received salvage treatment. The R-M regimen consisted of rituximab and MTX, while the R-MA regimen consisted of rituximab, MTX and Ara-C. Rituximab (375 mg/m2) was administered on day 0. HD-MTX (3.5 g/m2) was administered intravenously over 4 h on day 1. Patients received adequate pre-and post-MTX hydration, urinary alkalization and leucovorin rescue. Leucovorin rescue was started 24 h after completing HD-MTX infusion and administered at 15 mg every 6 h until serum MTX levels were ≤ 0.05 μmol/l. Ara-C (2 g/m2) was administered intravenously every 12 h on days 2–3. All patients who had grade 3–4 neutropenia with infection received prophylactic lenograstim after each cycle and the dose of Ara-C was reduced thereafter.

### Response evaluation

Treatment responses were evaluated every 2 induction chemotherapy cycles or at any time the neurological symptoms worsened or re-emerged by brain magnetic resonance imaging (MRI) with contrast enhancement. The responses were classified as complete remission (CR), unconfirmed complete remission (CRu), partial remission (PR), stable disease (SD), or progression disease (PD), according to the International Primary CNS Lymphoma Collaborative Group (IPCG) criteria^14^. For statistical analysis, CR and CRu defined by the IPCG criteria were combined into a single “CR” category previously defined by Response Evaluation Criteria in Solid Tumors in this study. Overall response = CR + PR. After completing the treatment, patients were assessed by repeated enhanced brain MRI every 3 months for the first 2 years and every 6 months for the next 3 years. OS was determined as the period from the date of diagnosis of PCNSLs to death from any cause or the last follow-up. PFS was defined as the time from diagnosis to disease progression or death due to PCNSL or last follow-up.

### Determination of length of hospital stay (LOS)

LOS was calculated from the day of chemotherapy to discharge and recorded in whole days. When a case was readmitted for side effects, the duration was added to the initial LOS. LOS was calculated for the R-M group and R-MA group, respectively.

### Cost analysis

The cost of hospital stays and antibiotics was determined from patient billing records and converted to US dollars at the currency exchange rate at the time of this study. The cost of hospital stays and antibiotics were calculated for the R-M group and R-MA group, respectively.

### Toxicity evaluation

Treatment toxicities were evaluated according to the National Cancer Institute Common Toxicity Criteria (CTCAE) version 4.0(https:// evs.nci.nih.gov/ftp1/CTCAE /CTCAE_ 4.03).

### Statistical analysis

Continuous variables were summarized as means with 95% confidence intervals (95% CIs) and compared with an unpaired t-test. Categorical variables were summarized as frequencies with associated percentages. The patient characteristics and treatment responses of the two therapeutic groups were compared using the chi-square test or Fisher’s exact test. The OS and PFS were calculated with the Kaplan–Meier method and compared with the log-rank test. The multivariate analysis for OS and PFS was performed based on the Cox proportional hazards regression model. The results were expressed as hazard ratios (HR) and their 95% CIs. Those factors with *p* values less than 0.1 in univariate analysis were used to construct multivariate models for OS and PFS. Landmark-analyses was used to minimize the bias in favor of responders who could survive long enough to have a response assessment. For continuous variables, the best-identified cutoff point was determined by receiver operating characteristic (ROC) curve analysis. All statistical analyses were performed using SPSS v17.0 for Windows (SPSS Inc, Chicago, IL). An alpha value of two-sided *p* < 0.05 was considered statistically significant.

### Ethics approval

This study was approved by the Human Ethics Committee of the Second Affiliated Hospital, School of Medicine, Zhejiang University, China.

### Consent to participate

Informed consent was obtained from all participants included in the study.

## Results

### Patient characteristics and treatment

A total of 34 patients receiving R-M regimen were enrolled. The diagnosis was achieved by surgery (40.5%), or stereotactic biopsy (59.5%), and no patients were diagnosed based on CSF. All PCNSLs were confirmed to be DLBCL. The median age of patients was 62 years (range, 27 to 80 years; 23 patients were > 60 years old, and 14 were ≤ 60 years old). Five patients (13.5%) had elevated LDH above the upper limit of normal. 77.1% (27/35) patients had non-germinal center B cell-like (non-GCB) DLBCL.

Fourteen patients receiving the R-MA regimen as induction chemotherapy were identified as the positive control group. The characteristics of the 51 patients are described in Table 1. These patients were treated according to their age, financial situation and willingness. The demographics and baseline clinical characteristics (sex, ECOG, LDH, deep structure involvement, biopsy type, Karnofsky performance status (KPS), CD10, C-Myc, Bcl-2, Bcl-6, MUM-1, pathological phenotype, and CSF protein) were not different between patients treated with the R-M and R-MA regimens, except for the age and number of lesions. Patients in the R-M group were older (*p* = 0.006) than the R-MA group, and more patients in the R-M group had multiple lesions (*p* = 0.024). No vitreoretinal lymphoma was identified through slit lamp examination by specialized ophthalmologist in both groups. Among the 34 out of 51 patients undoing routine CSF examination, no lymphoma cells were identified in CSF.

**Table 1:** Baseline patient characteristics and the distribution of lymphoma subtypes.

Factors		Total (n = 51)	R-M (n = 37)	R-MA (n = 14)	*p* value
Age (year)	(median, range)	60 (27–80)	62 (27–80)	56 (41–64)	0.012
	> 60 y	25 (49.0%)	23 (62.2%)	2 (14.3%)	
	≤ 60 y	26 (51.0%)	14 (37.8%)	12 (85.7%)	0.002
Gender	Male	28 (54.9%)	22 (59.5%)	6 (42.9%)	
	Female	23 (45.1%)	15 (40.5%)	8 (57.1)	0.29
Multifocal lesions	Yes	24 (47.1%)	21 (56.8%)	3 (21.4%)	
	No	27 (52.9%)	16 (43.2%)	11 (78.6%)	0.024
Deep-brain involvement	Present	31 (60.8%)	22 (59.5%)	9 (64.3%)	
	Absent	20 (39.2%)	15 (40.5%)	5 (35.7%)	0.75
Biopsy type	Surgical	21 (41.2%)	15 (40.5%)	6 (42.9%)	
	Stereotactic	30 (58.8%)	22 (59.5%)	8 (57.1%)	0.88
ECOG score	1	0 (0%)	0 (0%)	0 (0%)	
	2	26 (51.0%)	17 (45.9%)	9 (64.3%)	
	3	15 (29.4%)	12 (32.4%)	3 (21.4%)	
	4	10 (19.6%)	8 (21.6%)	2 (14.3%)	0.51
KPS score	< 70	32 (62.7%)	24 (64.9%)	8 (57.1%)	
	≥ 70	19 (37.3%)	13 (35.1%)	6 (42.9%)	0.61
LDH	Elevated	7 (13.7%)	5 (13.5%)	2 (14.3%)	
	Normal	44 (86.3%)	32 (86.5%)	12 (85.7%)	1.00
CSF protein	Elevated	22/34 (64.7%)	16/25 (64.0%)	6/9 (66.7%)	
	Normal	12/34 (35.3%)	9/25 (36.0%)	3/9 (33.3%)	1.00
C-Myc	Negative	53.3%	53.1%	53.8%	
	Positive	46.7%	46.9%	46.2%	0.96
Bcl-2	Negative	34.0%	35.3%	30.8%	
	Positive	66.0%	64.7%	69.2%	1.00
Bcl-6	Negative	20.4%	22.2%	15.4%	
	Positive	79.4%	77.8%	84.6%	0.71
CD10	Negative	77.6%	80.6%	69.2%	
	Positive	122.4%	19.4%	30.8%	0.40
MUM-1	Negative	14.6%	20%	0%	
	Positive	85.4%	80%	100%	0.17
Pathology phenotype	Non-GCB	35/48(72.9%)	27/35(77.1%)	8/13(61.5%)	0.28
	GCB	13/48(27.1%)	8/35(22.9%)	5/13(38.5%)	

*Notes* CSF, cerebrospinal fluid; ECOG, Eastern Cooperative Oncology Group; GCB, germinal center B-cell-like; KPS, Karnofsky performance status; LDH, lactate dehydrogenase; R-M, combination regimen of high-dose methotrexate and rituximab; R-MA, combination regimen of rituximab, high-dose methotrexate and cytarabine.

### Response and survival

**Figure 1:**
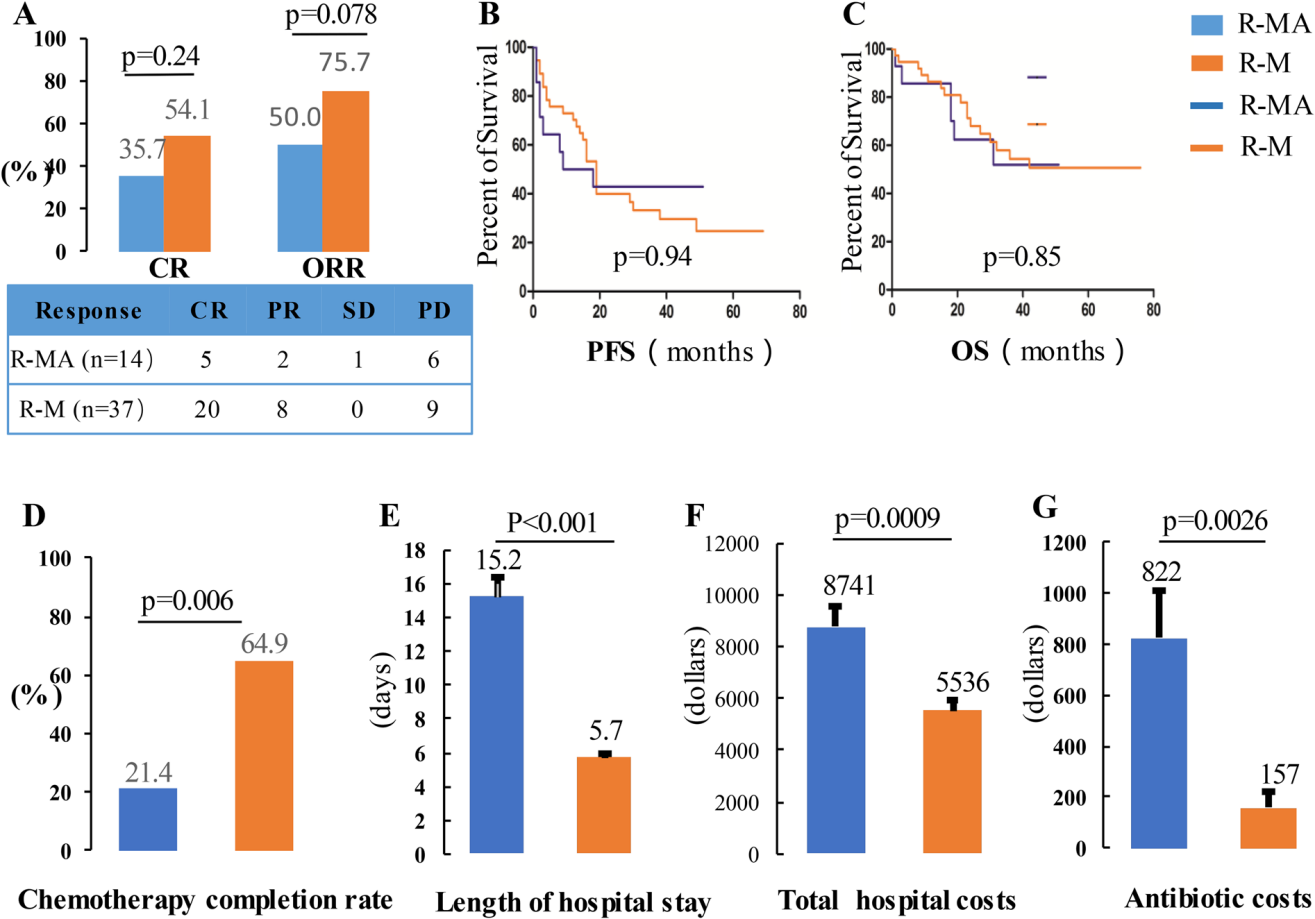
Response, survival and cost by treatment group. (**A**) Response; (**B**) Progression-free survival; (**C**) Overall survival; (**D**) Chemotherapy completion rate; (**E**) Average length of hospital stay; (**F**) Average total hospital cost per course per patient; (**G**) Average antibiotic costs per course per patient.

Thirty-seven patients received a total of 184 cycles of the R-M regimen with a median of 6 cycles per patient (range, 1–6). At the last follow-up, 20 achieved CR (54.1%), 8 achieved PR (21.6%), 0 had SD (0%), and 9 had PD (24.3%). Seven (18.9%) patients were consolidated with WBRT. Fourteen patients received a total of 39 cycles of the R-MA regimen, with a median of 3 cycles per patient (range, 1–6). At the last follow-up, 5 patients achieved CR (35.7%), 2 achieved PR (14.3%), 1 had SD (7.1%), and 6 had PD (42.9%). Four (28.6%) patients were consolidated with WBRT. The R-M regimen produced a comparable CR rate (54.1 vs. 35.7%, *p* = 0.24) and overall response rate (ORR) (75.6% vs. 50.0%, *p* = 0.078), compared with the R-MA regimen (Fig. 1A).

Follow-up data were available for all patients in both groups. The median follow-up duration was 30.0 months (range,1–76) in the R-M group, which was similar to 27.5 months (range, 1–51) in the R-MA group (*p* = 0.44). The median OS was not reached in either cohort with estimated 3-year OS rates of 54.4% and 51.9% in the R-M and R-MA group, respectively. The median PFS of the R-M group was 19.0 months (95% CI, 16.0–22.0) and the median PFS of R-MA group was 9.0 months (95% CI, 0–27.3). The estimated 2-year PFS rates were36.6% and 42.9% in the R-M and R-MA groups, respectively. The PFS (Fig. 1B).and OS (Fig. 1C) for the R-M group were not inferior to the R-MA group (*p* = 0.94, *p* = 0.85, respectively.).

### Toxicity and cost

Thirty-seven patients received a total of 184 cycles of the R-M regimen with a median of 6 cycles per patient (range, 1–6). Twenty-four of the 37 patients completed the entire protocol. Only 1 patient (2.7%) was withdrawn due to severe myelosuppression with infection and 9 patients (24.3%) were withdrawn due to disease progression. The remaining 3 patients were withdrawn due to personal reasons. Fourteen patients received a total of 39 cycles of the R-MA regimen, with a median of 3 cycles per patient (range, 1–6). Only three of the 14 patients completed the entire protocol. Indeed, five patients (35.7%) were withdrawn due to severe myelosuppression with infection, and six patients (42.9%) were withdrawn due to the disease progression. The total chemotherapy completion rate was higher in the R-M group than in the R-MA group (64.9 vs. 21.4%, *p* = 0.006) (Fig. 1D). The chemotherapy interruption rate due to toxicities was significantly lower in the R-M group than in the R-MA group (2.7 vs. 35.7%, *p* = 0.004).

The most frequent toxicities were hematologic toxicities and infection, as summarized in Table 2. The R-M group experienced significantly fewer frequent grade 3–4 hematological toxicities (3.4 vs. 59.0%, *p* < 0.001) and febrile neutropenia (2.7 vs. 41.0%, *p* < 0.001) than the R-MA group. Patients in the R-M group experienced significantly less frequent grade 3 neutropenia (1.6 vs. 15.4%, *p* = 0.001), grade 4 neutropenia (1.1 vs. 43.6%, *p* < 0.001), grade 3 anemia (0.5 vs. 12.8%, *p* = 0.001), grade 4 anemia (0 vs. 10.3%, *p* = 0.001) and grade 4 thrombopenia (1.1 vs. 51.3%, *p* < 0.001) than the R-MA group. Urinary tract infections (1.6 vs. 17.9%, *p* < 0.001) and pneumonia (1.6 vs. 17.9%, *p* < 0.001) were also observed much less frequently in the R-M group than in the R-MA group. Other nonhematological toxicities were generally mild to moderate and reversible and occurred at similar frequencies across both treatment groups. No treatment-related deaths were observed in either group. There were 2 cases of atrial fibrillation, one case of deep vein thrombosis, one case of acute heart failure, and one case of grade 4 skin rash in the R-MA group.

**Table 2:** Main adverse effects by treatment group.

Toxicities	R-M (n = 184 cycles)	R-MA (n = 39 cycles) cecs)	*p* value
Grade 3 neutropenia	3(1.6%)	6(15.4%)	0.001
Grade 4 neutropenia	2(1.1%)	17(43.6%)	< 0.001
Grade 3 thrombopenia	4(2.2%)	3(7.7%)	0.10
Grade 4 thrombopenia	2(1.1%)	20(51.3%)	< 0.001
Grade 3 anemia	1(0.5%)	5(12.8%)	0.001
Grade 4 anemia	0(0%)	4(10.3%)	0.001
Febrile neutropenia	5(2.7%)	16(41.0%)	< 0.001
Urinary tract infection	3(1.6%)	7(17.9%)	< 0.001
Pneumonia	3(1.6%)	7(17.9%)	< 0.001
Oral candidiasis	3(1.6%)	0(0%)	1.00
Sepsis	1(0.5%)	1(2.6%)	0.32
Acute infectious enteritis	1(0.5%)	0(0%)	1.00
Herpes zoster	1(0.5%)	0(0%)	1.00
Grade 3 Hepatotoxicity	3(1.6%)	3(7.7%)	0.068
Grade 4 Hepatotoxicity	1(0.5%)	0(0%)	1.00
Creatinine elevated	2(1.1%)	0(0%)	1.00
Deep vein thrombosis	0(0%)	1(2.6%)	0.18
Atrial fibrillation	0(0%)	2(5.1%)	0.03
Acute heart failure	0(0%)	1(2.6%)	0.18
Grade 4 skin rash	0(0%)	1(2.6%)	0.18

*Notes* R-M, combination regimen of high-dose methotrexate and rituximab; R-MA, combination regimen of rituximab, high-dose methotrexate and cytarabine.

The average LOS was 5.7 days (95% CI 5.3–6.1) in the R-M group, which was shorter than 15.2 days (95% CI 12.7–17.3) observed in the R-MA group (*p* < 0.001) (Fig. 1E). The mean total hospitalization cost per cycle of the R-M regimen per patient was $5536 (95% CI 4830–6454), with a mean total antibiotic cost per cycle per patient of $157 (95% CI 37–291). The mean total hospitalization cost per cycle of the R-MA regimen per patient was $8741 (95% CI 6904–10,352), with a mean total antibiotic cost per cycle per patient of $822 (95% CI 417–1185). The mean total hospitalization cost (Fig. 1F). and mean total antibiotic cost per cycle per patient (Fig. 1G) in the R-MA group were 1.58 times (*p* = 0.0009) and 5.24 times (*p* = 0.0026) that of the R-M group, respectively.

### Univariate and multivariate analyses of prognostic indicators

**Figure 2:**
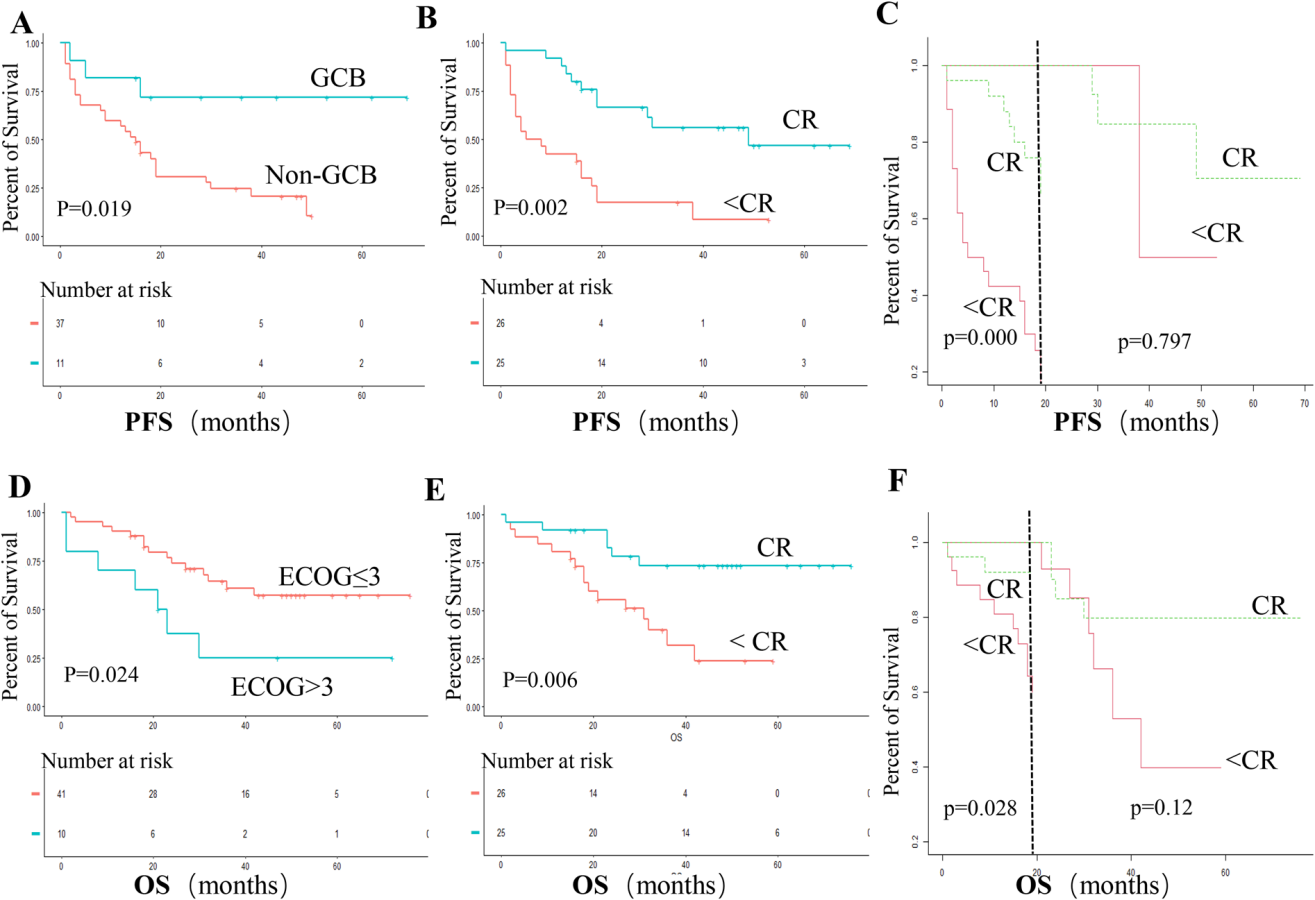
Kaplan–Meier survival curves and landmark analysis. (**A**) Progression-free survival by pathological subtype (GCB vs. non-GCB); (**B**) Progression-free survival by response (CR vs. not CR) at the end of induction chemotherapy; (**C**) Landmark analysis of progression-free survival by response (CR vs. not CR) at the end of induction chemotherapy; (**D**) Overall survival by subtype (ECOG > 3 vs. ECOG ≤ 3); (**F**) Overall survival by response (CR vs. not CR) at the end of induction chemotherapy; (**E**) Landmark analysis of overall survival by response (CR vs. not CR) at the end of induction chemotherapy.

To identify the prognostic indicators, all 51 patients were enrolled in the univariate and multivariate analyses. The results of univariate and multivariate analyses of prognostic indicators are summarized in Table 3. The univariate analysis indicated that GCB subtype (*p* = 0.019, Fig. 2A), WBRT consolidation (*p* = 0.033), and CR at the end of the induction therapy (*p* = 0.002, Fig. 2B) were associated with increased PFS. While age > 60 (*p* = 0.041), ECOG > 3 (*p* = 0.024, Fig. 2D), and CR at the end of the induction therapy (*p* = 0.006, Fig. 2E) were associated with increased OS. Disappointingly, we did not observe any association of LDH or deep brain involvement with PFS or OS. We incorporated all those factors with *p* values < 0.1 in univariate analysis into the multivariate Cox analysis revealed that GCB subtype(*p* = 0.014) and CR at the end of induction chemotherapy (*p* = 0.009) were identified as dependent prognostic indicators for PFS. While ECOG > 3 (*p* = 0.025), and CR at the end of induction chemotherapy (*p* = 0.002) were independent prognostic factors for OS. To further delineate the impacts of end-of-induction response on survival, we performed additional landmark analysis with landmark time at 19 months (median PFS time). This analysis showed CR at the end of induction chemotherapy were associated with better PFS (Fig. 2C) and OS (Fig. 2F) only in the early survival time. Moreover, cox analysis indicated CR at the end of induction chemotherapy was a dependent prognostic indicator for OS both in early period and late period of survival time.

**Table 3:** Univariate and multivariate analyses of overall and progression-free survival.

Variable	No. event /total	PFS				No. event /total	OS			
		Univariate analysis		Multivariate analysis			Univariate analysis		Multivariate analysis	
		HR(95% CI)	*P* value	HR(95% CI)	*p* value		HR (95% CI)	*p* value	HR (95% CI)	*p* value
Age > 60y	17/25	1.47(0.71–3.05)	0.299			13/25	2.83(1.04–7.69)	0.041	2.56(0.91–7.21)	0.074
Deep-brain involvement	20/31	1.11(0.55–2.25)	0.755			13/31	0.90(0.38–2.11)	0.805		
ECOG > 3	9/10	1.94(0.89–4.20)	0.095	1.80(0.76–4.29)	0.183	7/10	2.83(1.15–7.00)	0.024	3.14(1.16–8.54)	0.025
Elevated LDH	5/7	1.12(0.53–2.38)	0.764			4/7	1.73(0.58–5.12)	0.323		
Multiple focal	18/24	1.84(0.92–3.66)	0.083	1.42(0.63–3.21)	0.399	12/24	1.49(0.64–3.45)	0.353		
Cytarabine treatment	8/14	0.97(0.44–2.16)	0.940			6/14	1.09(0.43–2.80)	0.853		
WBRT consolidation	5/11	0.35(0.14–0.92)	0.033	0.39(0.12–1.16)	0.088	3/11	0.38(0.11–1.28)	0.118		
GCB subtype	3/11	0.24(0.07–0.79)	0.019	0.21(0.06–0.73)	0.014	2/11	0.28(0.07–1.21)	0.089	0.30(0.07–1.31)	0.109
CR at the end of treatment	11/25	0.32(0.15–0.66)	0.002	0.35(0.16–0.76)	0.009	6/25	0.26(0.11–0.74)	0.006	0.20(0.07–0.55)	0.002
≤ 19 months	11/25	0.22(0.10–0.50)	0.000	0.32(0.14–0.76)	0.01	6/25	0.18(0.04–0.83)	0.028	0.17(0.04–0.80)	0.025
> 19 months	3/12	0.74(0.08–7.25)	0.797	1.23(0.11–13.7)	0.86	2/20	0.36(0.10–1.29)	0.12	0.17(0.03–0.93)	0.041

*Notes* CI, confidential interval; CR, complete remission; HR, hazard ratio; ECOG, Eastern Cooperative Oncology Group; GCB, germinal center B-cell-like; LDH, lactate dehydrogenase; OR, overall remission; OS, overall survival; PFS, progression-free survival.

## Discussion

We retrospectively evaluated the efficacy, toxicity and cost of the R-M regimen, and compared it with the R-MA regimen. We found that the response rates and survival were not significantly different between the two regimens, indicating that the efficacy of the R-M regimen was comparable to the R-MA regimen. However, the R-M regimen yielded a higher chemotherapy completion rate with fewer severe toxicities, shorter LOS and lower cost than the R-MA regimen for patients with newly diagnosed PCNSLs.

Induction chemotherapy based on HD-MTX is considered as the standard approach for newly diagnosed PCNSLs. However, the best chemotherapeutic regimen remains unclear. Combinations with other chemotherapeutic agents have been shown to improve response and survival in patients with PCNSL, although few randomized clinical trials have supported this practice^5^. However, comparisons were still challenging due to differing treatment schedules, consolidation strategies, and dosages and patient heterogeneity between the studies. It seems that adding more drugs into the HD-MTX-based chemotherapy regimens tends to improve the outcome but brings more toxicities. Even with the conflicting results of rituximab in two prospective randomized studies, the relatively low toxicities and single-agent activity of rituximab in relapsed/refractory PCNSLs have resulted in its widespread use. Therefore, among these combination regimen, R-M regimen appears to be the combination chemotherapy with the least additional toxicities. Holdhoff et al.^2^ revealed that compared with the single HD-MTX group, the R-M regimen improved CR rates and survival. In contrast, Kansara et al.^3^ reported that the addition of Rituximab to HD-MTX did not appear to improve outcomes. Both studies indicate that the addition of rituximab was feasible and well tolerated with overall minimal added toxicities. Our data suggest that the efficacy of the R-M regimen is comparable to that of the R-MA regimen, and most importantly, the R-M regimen shows the advantage of a better toxicity profile and less burden for health systems, although the sample size of the current study was small.

The combination of HD-Ara-C with HD-MTX has significantly improved outcomes, as described in large studies^4,15^. After the IELSG20 results were first published in 2009^4^, HD-Ara-C was widely applied in the management of PCNSLs. Recently, several studies reported the disappointing results of the HD-Ara-C containing regimens. One retrospective study demonstrated that neither PFS nor OS was higher in the Ara-C group than a therapeutic regimen without Ara-C^16^. A randomized clinical trial(RCT) by Wu et al.^17^ showed that the non-Ara-C group (receiving the fotemustine, teniposide, and dexamethasone (FTD) regimen) had better apparent effects and safety than the Ara-C group (receiving the MA regimen). Hematological toxicities were the main concern. As expected, grade 3–4 hematological toxicities including neutropenia (90%, 56% and 59%, respectively), thrombocytopenia (92, 74 and 59%, respectively) and anemia (46%, 36% and 23%, respectively) were quite common in the IELSG20 trial, IELSG32 trial and the current study. Chemotherapy interruption due to toxicities occurred in 17.9%, 12% and 35.7% of patients in the IELSG20, IELSG32 and the current study, respectively. What’s worse, a treatment-related mortality of 6–9% was observed in these clinical trials. Thus, more effective and less toxic therapeutic regimens are urgently needed to balance therapy intensification with side effects, especially for old and weak patients. Our data suggest that the efficacy of the R-M regimen is comparable to that of the R-MA regimen with much fewer toxicities. We considered that the high frequency of grade 3–4 side effects with Ara-C might compromise the OS benefit. These toxicities were the main contributors to cost and prolonged hospital stay, resulting in a financial burden. In recent years, newly developed targeted drugs may provide new treatment options in PCNSL patients^18^. Ibrutinib (a selective covalent BTK inhibitor)^19^, nivolumab^20^ (a PD1 block) and lenalidomide (an immunomodulatory imide drug)^21^, showed promising response rates in refractory/recurrent PCNSL patients with good tolerability. we initiated a phase-2 trial, comparing the efficacy and safety of the R-M regimen with or without lenalidomide for newly-diagnosed PCNSLs, is ongoing (NCT04481815). As a result, we believe that in the era of rituximab and new targeted drugs, the role of HD-Ara-C in the induction chemotherapy for PCNSLs needs to be reassessed, which warrants further evaluation in randomized trials.

There are two prognostic scoring systems developed specifically for PCNSLs. One is the International Extranodal Lymphoma Study Group (IELSG) scoring system, which divides patients into three different risk classes based on age, ECOG performance status, LDH, CSF total protein concentration, and involvement of deep brain structures^22^. The other is the Memorial Sloan-Kettering Cancer Center (MSKCC) scoring system, which divides patients into three different risk classes, using age and KPS^23^. Although the prognostic value of most factors remains controversial, performance status and age are the most consistent prognostic factors for PCNSLs. In our retrospective analysis, ECOG > 3 was predictive of prognosis in multivariate analyses. In systemic DLBCL, the GCB subtype is associated with a better prognosis than the non-GCB subtype^24^. However, multiple previous studies reported no significant difference in survival between patients with GCB and those with non-GCB of PCNSLs^25,26^. In contrast to previous studies, we found that none of the 11 patients in the GCB group died by the last follow-up and GCB was associated with increased PFS in both the univariate analyses and multivariate analyses. Interestingly, we found that end-induction response could be used for patient prognostication. Patients who did not achieve CR at the end of induction treatment had a poor OS compared with those who did, indicating that treatment should be resumed for those without CR with consolidation (such as WBRT or autologous bone marrow transplantation) at the end of induction treatment^27,28^.

Our study has several limitations given that it is a retrospective, single-institution analysis with modest sample size. First, the CSF protein data of 33% of patients were not available for the current study, similar to other retrospective studies. Therefore, it was impossible to determine different responses among IELSG subgroups. Second, it should be highlighted that only 3 of the 14 enrolled patients (21.4%) in the R-MA group completed the entire protocol, which limited true judgments of its effectiveness. Third, there was an unusual high proportion of early progression events (42.9%) in the R-MA arm, which may be due to the higher ECOG performance scores in this real-world study. However, our study has strengths. All our patients had a uniform, identical pathological diagnosis and received standardized treatment schedules, despite this being a retrospective study.

## Conclusion

In conclusion, the efficacy of the R-M regimen is comparable to that of the R-MA regimen, but with fewer toxicities, well tolerability, shorter LOS and lower cost. Our results suggest that R-M regimen is an effective and cost-effective combination treatment for PCNSLs, which warrants further evaluation in randomized trials.

## Data Availability

All data generated or analyzed during this study are included in this published article.
